# The Biobehavioral Family Model: An Evidence-Based Approach to Biopsychosocial Research, Residency Training, and Patient Care

**DOI:** 10.3389/fpsyt.2021.725045

**Published:** 2021-10-05

**Authors:** Beatrice L. Wood, Sarah B. Woods, Sourav Sengupta, Turya Nair

**Affiliations:** ^1^Department of Psychiatry, Jacobs School of Medicine and Biomedical Sciences, University at Buffalo, Buffalo, NY, United States; ^2^Department of Pediatrics, Jacobs School of Medicine and Biomedical Sciences, University at Buffalo, Buffalo, NY, United States; ^3^Department of Family and Community Medicine, University of Texas Southwestern Medical Center, Dallas, TX, United States

**Keywords:** biobehavioral model, biopsychosocial model, child psychiatry, family practice, family relations, graduate medical education

## Abstract

Engel's biopsychosocial model, based in systems theory, assumes the reciprocal influence of biological, psychological, and social factors on one another and on mental and physical health. However, the model's application to scientific study is limited by its lack of specificity, thus constraining its implementation in training and healthcare environments. The Biobehavioral Family Model (BBFM) is one model that can facilitate specification and integration of biopsychosocial conceptualization and treatment of illness. The model identifies specific pathways by which family relationships (i.e., family emotional climate) impact disease activity, through psychobiological mechanisms (i.e., biobehavioral reactivity). Furthermore, it is capable of identifying positive and negative effects of family process in the same model, and can be applied across cultural contexts. The BBFM has been applied to the study of child health outcomes, including pediatric asthma, and adult health, including for underserved primary care patients, minoritized samples, and persons with chronic pain, for example. The BBFM also serves as a guide for training and clinical practice; two such applications are presented, including the use of the BBFM in family medicine residency and child and adolescent psychiatry fellowship programs. Specific teaching and clinical approaches derived from the BBFM are described in both contexts, including the use of didactic lecture, patient interview guides, assessment protocol, and family-oriented care. Future directions for the application of the BBFM include incorporating temporal dynamics and developmental trajectories in the model, extending testable theory of family and individual resilience, examining causes of health disparities, and developing family-based prevention and intervention efforts to ameliorate contributing factors to disease. Ultimately, research and successful applications of the BBFM could inform policy to improve the lives of families, and provide additional support for the value of a biopsychosocial approach to medicine.

## Introduction

In his 1977 paper, Engel stated his belief that in order for a medical science to have a complete understanding of disease, as the underpinning for rational treatment and health care, “it would need to incorporate the patient, the social context in which he lives, and the complementary system devised by society to deal with the disruptive effects of the illness, that is the physician role and the health care system” ((1), p.132). In short, medicine needed a *biopsychosocial model* (BPS) [See (2) for an in-depth analysis of Engel's Biopsychosocial Model in the context of paradigms, models, and theories]. Engel's Biopsychosocial Model was based in systems theory, which assumes that all levels of organization are linked to each other in a hierarchical relationship, so that change in one affects change in the others. Thus, the model assumed that biological, psychological, and social factors were interrelated and influenced one another in both physical and mental disease. However, the BPS model itself did not include specific aspects of the biological, psychological, or social factors, nor mechanisms by which inter-relation occurs (3). This lack of specificity limited critical research which would fully instantiate the model and provide guidance for adequate scientific inquiry, education, and clinical applications of the model.

## The Biopsychosocial Continuum of Disease

**Figure 1:**
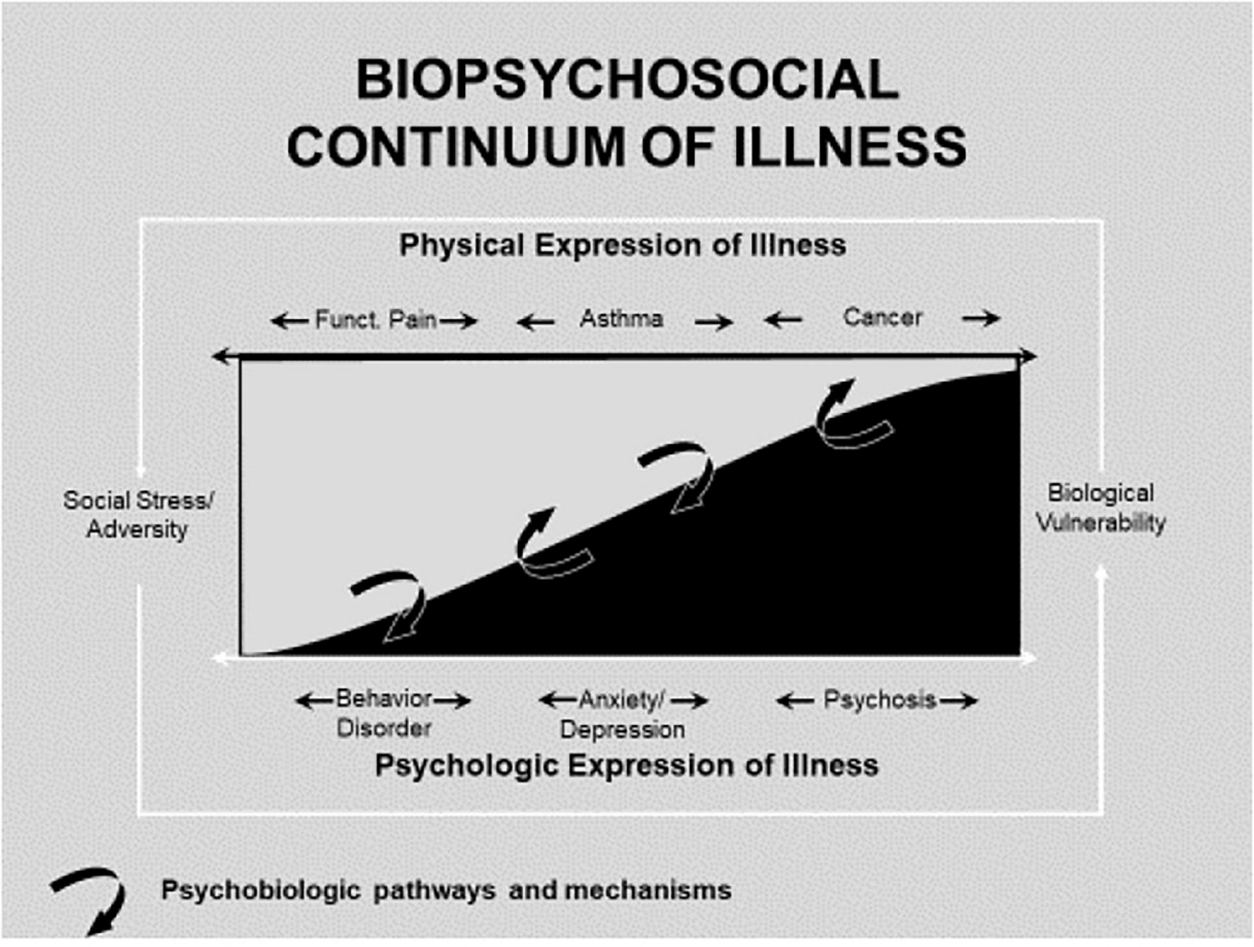
Biopsychosocial continuum of illness [reformulated from Miller and Wood (6)].

Biopsychosocial conceptualization has been enhanced by recent advancements in neuroscience with regard to specificity of the rich interplay of social stress, psyche, and soma in both physically and emotionally manifested illness (4). Research has also demonstrated the powerful role that social stress plays in emotional and physical illness (5). These scientific advances can be organized into the following heuristic framework conceptualizing the relations among physical and psychological aspects of illness [Figure 1, reformulated from (6)]. This framework illustrates the interplay of social, psychological, and biological manifestation of illness, and the pathways, and mechanisms (curved arrows) underlying these causal effects. The framework rests firmly upon the assumption that there are verifiable psychobiological mechanisms that mediate the interplay of social stress and adversity with psychological and biological factors to determine psychologic/emotional and physical illness (6). Using this framework will facilitate identification and verification of mechanisms and pathways in order to fully understand the biopsychosocial aspects of disease, and to optimally target interventions.

## The Biobehavioral Family Model

**Figure 2:**
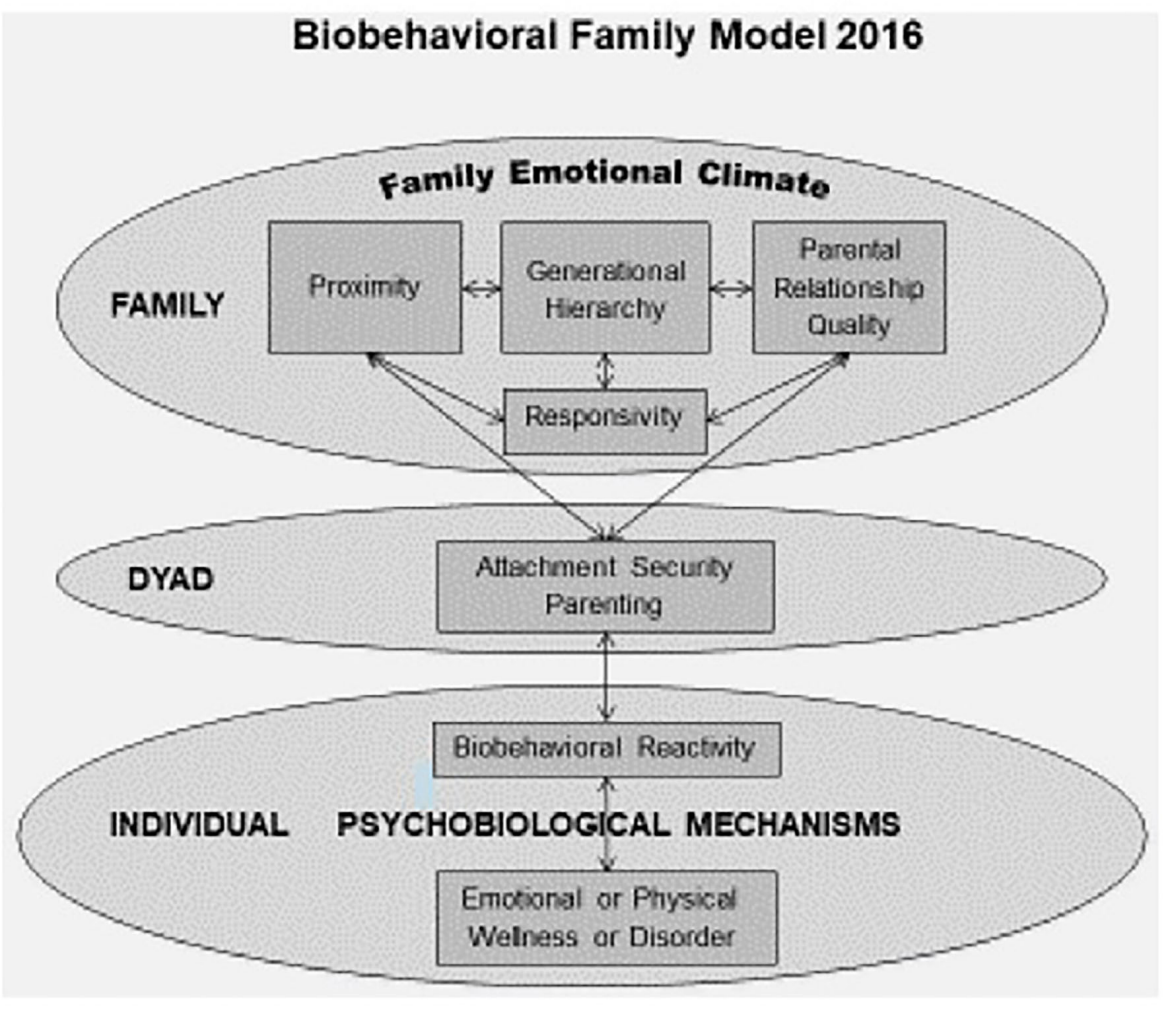
The biobehavioral family model (7).

One obstacle to integrated biopsychosocial care is the lack of integrative models that provide a shared language and conceptualization across disciplines. The Biobehavioral Family Model (BBFM) (7) is one model that can facilitate specification and integration of biopsychosocial conceptualization and treatment of illness (see Figure 2). It is one of many possible biopsychosocial models. However, the BBFM prioritizes family factors based on the evolutionary assumption that the family social system serves as a buffer and means of adapting to social stress and adversity. Thus, to the extent that the family relations are functional, the family will buffer individual family members from stress; but, if family relations are dysfunctional they may exacerbate the effects of stress and adversity on an individual family member's health. The BBFM was developed to identify specific pathways by which family relations impact both emotional/psychological and physical illness, through psychobiological pathways. The value of the BBFM is to facilitate the development of knowledge through research and apply it to training procedures, to the practice of family-based interventions, and, eventually, to family policy.

There are three aspects of the model which together are an advance in family theory. First, the BBFM is based in normative, rather than in dysfunctional concepts of family relations, so it is capable of identifying positive aspects of family relational process. Second, the model is dimensional. This means that each of its family relational constructs is conceived as a quantitative continuum. Family relational process characterized by the positive ends of the continua would suffer the effects of stress (internal and external) on the individual, whereas family process characterized by the negative ends of the continua would transmit internal family stress and exacerbate external stress on the individual family member. These two aspects provide for consideration of *both* protective and negative effects of family relational process in the same model. Finally, the constructs of the BBFM are not culturally bound, so it can be applied across ethnic groups and social class. A comprehensive presentation of the BBFM is found elsewhere (7).

The BBFM model originally focused on the child. However, the model can be, and has been, adapted to study patients across the lifespan and in various SES and ethnic groups. Explorations are underway regarding its value as a model of family influences on resilience. The BBFM incorporates specific conceptual dimensions of family relational process: (1) family emotional climate; (2) interpersonal proximity; (3) generational hierarchy; (4) parent-parent relationship quality; (5) responsivity; (6) attachment security; and (7) biobehavioral reactivity (7).

### Family Emotional Climate

Family emotional climate refers to the overall intensity and valence of family emotional exchange. It colors all aspects of family relationships, and therefore is likely a key factor contributing to emotional status and outcomes in family members. A negative family emotional climate (NFEC) includes hostility, criticism, verbal attacks, etc., and it is similar to the criticism construct of Expressed Emotion [EE; (8)]. Positive aspects include warmth, affection, support, affirmation, etc. Family emotional climate is characterized by the intensity and balance of this negative and positive emotional exchange among family members. This balance or imbalance can be construed as reflecting one aspect of family-level emotion regulation or dysregulation.

### Proximity

Proximity is an index of interpersonal connectedness, based on the extent to which family members share physical affection, private information, and emotions (9, 10).

### Generational Hierarchy

Generational hierarchy refers to the extent to which caregivers are in charge of the children by providing nurturance, guidance, and limit setting through strong parental alliance and absence of cross-generational coalitions (9, 10).

### Parent-Parent Relationship Quality

Parent-parent relationship quality refers to interaction patterns, which include mutual support, understanding, and adaptive disagreement (respectful and resolving) vs. hostility, rejection, and conflict. Parent-parent relationship quality sets the stage for family level emotional climate. It also has direct effects on children's emotional functioning (11), with emotional security mediating the link (12). Parental conflict is accompanied by physiologic stress responses in the exposed child (13, 14).

### Responsivity

Responsivity refers to the extent to which family members are behaviorally, emotionally, and physiologically responsive to one another. Moderate levels of emotional/physiological responsivity allow for empathic response among family members. Extremely high levels of responsivity can exacerbate maladaptive emotional/physiological resonance in the family, possibly worsening stress-influenced emotional or physical disorders. Extremely low levels of responsivity result in neglect or avoidance, leaving family members unbuffered from internal, familial, or environmental stressors. Family-wide levels of responsivity reflect family level emotion or stress regulation or dysregulation. Thus, family level emotion regulation and individual biobehavioral reactivity (see below) are inter-related.

### Attachment

Attachment refers to the biologically based, lifelong tendency of human beings under conditions of stress to seek, and receive some form of proximity (physical or emotional) with specific other persons who are perceived as protective or comforting, such that one's emotional and physiological disequilibrium are restored (15, 16).

### Biobehavioral Reactivity

Biobehavioral reactivity, the pivotal construct that links psychological to biological processes in the BBFM, is conceptualized as the degree or intensity with which an individual family member responds physiologically, emotionally, and behaviorally to stressors or emotional stimuli (17–20). Biobehavioral reactivity is tightly linked to emotion regulation and dysregulation because it is the phenomenological outcome of the convergence of the biopsychosocial processes inherent in stress and emotion regulation and dysregulation. Chronic levels of child emotion dysregulation are expressed as child depression and anxiety (21).

Emotion regulation is accompanied by a relatively stable physiological regulation, whereas emotion dysregulation is accompanied by physiological dysregulation. Emotion regulation buffers, while emotion dysregulation transmits (or escalates) the effect of stress and emotional challenge to disease processes by way of psychophysiological pathways. Thus, biobehavioral reactivity reflects the ability of the individual to regulate the physiological and behavioral aspects of stress and emotion. All aspects of the neurophysiological stress response system (autonomic, hypothalamic pituitary adrenal, neuroendocrine systems) are underlying biological pathways and mechanisms of biobehavioral reactivity (4).

### Psychobiologic Pathways

In order for the BBFM to be a viable model to explain the impact of family on medical disease, there must be viable *psychobiologic pathways and mechanisms* by which negative family relational process cascades to biobehavioral reactivity and thus impacts disease. Several studies have identified neuro-endocrine-immune stress pathways implicated in the impact of stressors on medical disease (4). In the case of the impact of family relational stress on child asthma, it has shown that child depression, which is evoked by negative family climate and insecure attachment, is associated with autonomic dysregulation (specifically a predominance of parasympathetic/cholinergic over sympathetic reactivity to stress). Since airway constriction in asthma is partially mediated by cholinergic pathways, such predominance of cholinergic reactivity results in airway compromise in response to laboratory stress (6, 22, 23). Other studies have shown that chronic family stress impacts child asthma by way of immune pathways, specifically by altering asthma-relevant cytokine and cortisol function (24).

The BBFM model is designed to be an empirically testable, refutable model. It has proven to be usefully modifiable and widely adaptable to various age groups and ethnic backgrounds. By design, the BBFM can address family effects which either protect from, or contribute to, physically and/or psychologically/emotionally manifested disease, and identify psychobiological mediators of these effects. Intentionally, the nature of the basic framework of the model lends itself to alteration and development.

One modification is to examine the effect of parental depression on child emotional and asthma disease activity, mediated by negative parenting and child depression. In a study of depressed mothers, Lim et al. showed that maternal depression predicted negative parenting which impacted child depression, which in turn predicted worse child asthma (25). In a study of two-parent families, parental depression predicted inter-parental hostility, which predicted negative parenting, child depression, and worse asthma disease activity (26). Another study showed that single maternal caregiver depression impacted child asthma mediated by both insecure attachment and child depression (Author).

## Pediatric Research in Support of the BBFM—Children With Asthma

The BBFM pathway from negative family climate to insecure attachment to depression (i.e., biobehavioral reactivity) was tested in a laboratory-based family interaction study of children with asthma. Structural equation modeling (SEM) demonstrated that NFEC (operationally defined as a predominance of negative/hostile over warm interactions) predicted child asthma disease severity, mediated by insecure attachment and child depression (27). Structural equation modeling focusing on two-parent families, using the same database, showed that parent-parent negative emotional climate predicted increased child asthma disease activity mediated by negative parenting (operationally defined as a predominance of negative/hostile over warm interactions) and child anxiety and depression (26). Thus, the results of these studies are suggestive that the BBFM may be useful in specifying family-psycho-biological pathways by which family relational stress impacts child physical well-being and disease. Furthermore, the findings suggest that the BBFM, as a dimensional model, may be used to examine how family function may buffer the impact of social stress on child asthma by examining the effects of the family configurations constituted by family relational patterns at the positive ends of the BBFM dimensions (i.e. when warmth predominates over negative/hostile interactions).

## BBFM Applied to Adult Health

The BBFM was originally developed to address pediatric illness. Subsequently, it has been adapted by Sarah Woods to examine the specific pathways by which family relationships impact adult health and illness. In the BBFM adaptation for adult health, family emotional climate is defined as before, but the impact of the intimate partner emotional climate is distinguished from the emotional climate of other relationships in the family (i.e., non-intimate family relationships). That is to say, the emotional climate in the adult intimate relationship is distinguished from the overall emotional climate of the patient's relationships with other family members.

The first test of the BBFM with an adult sample explored the ability of the model to predict the effects of family emotional climate on the physical health of underserved, primary care patients [aged 18–65; (28)]. The authors found that family emotional climate (measured as family functioning) was linked to disease activity (operationalized as illness symptoms and role limitations due to physical health) via biobehavioral reactivity, specifically depression, and anxiety symptoms. In this same study, a second model operationalized family emotional climate as romantic relationship satisfaction and found that biobehavioral reactivity served as a significant link between this measure of emotional climate and disease activity [expanded to include measures of general health and pain (28)]. This project supported the application of the model to adult family members, and research in this area has since flourished.

While application of the BBFM for adults has not incorporated parent-child attachment security as an additional mediator, there have been two extensions of the operationalization of family emotional climate to include parent-adult child relationships. First, Priest et al. (29) tested the contributions of adverse childhood experiences, including abuse and neglect experienced in the family during youth, alongside measures of concurrent intimate partner emotional climate and (non-intimate) family emotional climate, on self-rated health, comparative health, morbidity, and number of prescription medications as measures of disease activity. Second, recent research has operationalized family emotional climate to include maternal and paternal affection experienced during childhood, predicting health appraisal, and number of chronic conditions over 20 years (30). Expanding the definition of the family emotional climate construct to intentionally incorporate the emotional climate of family relationships experienced in childhood, intimate partner relationship quality, and concurrent non-intimate family emotional climate (including parents' relationships with their own children) constitute clinically relevant extensions of the BBFM explanatory model.

Both pediatric and the adult BBFMs posit, and test, biobehavioral reactivity as a critical mechanism linking the effects of emotional climate on health. Biobehavioral reactivity is operationalized as emotion dysregulation (e.g., anxiety and depression) for both models, and is linked to disease activity via the impact of psychophysiological stress reactions. However, research in adults has extended the operational definition of biobehavioral reactivity to include allostatic stress pathways, with allostatic load (31) as an index of biobehavioral reactivity [e.g., (32)]. In addition, the model has been extended to test the contribution of health behaviors, influenced by family emotional climate and in concert with biobehavioral reactivity, to impact adult health outcomes [e.g., (33)].

Many studies have now substantiated the role of both intimate partner relationships as well as non-intimate family relationships in understanding the impact of family on health for adults [e.g., (33, 34)]. Though both operationalizations of family emotional climate—the positivity or negativity, and intensity, of intimate partnerships *and* of non-intimate family relationships—have been significantly linked to disease activity in tests of the BBFM, studies have tended to support more powerful links for non-intimate family relationships, especially when negative and intense [e.g., (29, 35, 36)]. In addition, an application of the BBFM tested with a national, representative, epidemiological U.S. sample operationalized family emotional climate as marital strain and family support, while testing social support received from friends as an additional, contrasting factor (37). The former two measures (assessed as demanding, critical, unreliable, or irritating partner behaviors, and being able to rely on and open up to relatives, respectively) were supported as operationalizations of family emotional climate and were linked to disease activity via biobehavioral reactivity. Friends' support, however, was not significantly associated with health, directly nor indirectly. This finding highlights an advantage of the BBFM over more general models of relationships and health: the construct of “social support” remains loosely and variably defined; this lack of specificity often interferes with replication and application of these findings, and overlooks the specific and powerful impacts of family relationships. The BBFM lends the specificity necessary in order to develop family relational targets for intervention.

Woods et al. (30) subsequently expanded the concept of family emotional climate. Specifically, the authors found four distinct categorizations of family emotional climate, i.e., positive, negative, ambivalent, and indifferent climates, each predicting different outcomes. Their models indirectly linked a NFEC (marked by high strain, low support, and low parental affection) with worse disease activity (i.e., health appraisal) 20 years later via biobehavioral reactivity (i.e., negative affect reactivity reported via daily diary reports) at 10 years. Further, an ambivalent family emotional climate (marked by high strain plus high support) was directly linked to greater morbidity two decades later.

### Expanding the Construct of Biobehavioral Reactivity

Woods expanded the concept of biobehavioral reactivity beyond depression and anxiety for adults [reflective of emotion dysregulation via both affective and physiological symptoms; (29)] to include negative affect, defined as subjective distress and negative emotional states such as nervousness, irritability, fear, and frustration (38). Research also suggests physiological changes, such as cortisol and C-reactive protein response, and cardiovascular reactivity, for example, are components of negative affect reactivity (39, 40). Recent BBFM research used daily diary reports of negative affect related to stress exposure as an operationalization of biobehavioral reactivity. Specifically, Woods et al. (30) incorporated participants' reports of the frequency of 14 negative emotional states (e.g., restless, hopeless, lonely, ashamed) in response to specific stressors (e.g., arguments, work or school stress, discrimination) across 8 days into a test of the BBFM, finding that negative affect reactivity significantly mediated the link between a NFEC and disease activity (i.e., health appraisal) 20 years later.

Woods and colleagues have also incorporated tests of allostatic load as measures of biobehavioral reactivity conveying the effect of family emotional climate on disease activity for adults [e.g., (29)]. Priest et al. (32) found support for a broad-spectrum index of allostatic load in a cross-sectional test of the BBFM, whereby a NFEC (but not a negative intimate partner emotional climate) was significantly associated with disease activity (i.e., morbidity, prescription medication use) via both depression and anxiety, as well as allostatic load (comprised of indices of cardiovascular health, metabolic lipids, metabolic glucose, inflammation, and parasympathetic nervous system functioning). However, whereas Woods et al. (30) found support for negative affect reactivity as an operationalization of biobehavioral reactivity, the authors did not find support for allostatic load as a mediating pathway in their longitudinal test of the BBFM. It is possible that more fine-tuned quantifications of biological aging—patient-level measures of aging that compare aging adults' biomarker results to peer populations—may be more attuned to capturing variation in psychobiological pathways impacted by stress than static measures of allostatic load (41, 42).

Lastly, though not theorized as a pathway to health in the pediatric BBFM, tests of the model with adults have incorporated health behaviors as a potential additional mediator, alongside biobehavioral reactivity. Though emotional climate retains significance as a pathway to disease via stress reactivity, health behaviors have been tested as an additional pathway through which emotional climate affects physical health—and, as a variable that is correlated to (and impacted by) biobehavioral reactivity. In other words, the valence and intensity of relationship quality for adults has the potential to both discourage (or support) healthy health behaviors, as well as to potentiate (or decrease) stress reactivity. Thus, variation in adults' biobehavioral reactivity is theorized to covary with (to impact and be impacted by) health behaviors, contributing in turn to disease activity. Initial tests have found some support for the addition of health behaviors to the model: Roberson et al. (33) found stress-eating and exercise (both reported 10 years post-baseline) each served as significant links between baseline intimate partner emotional climate (i.e., marital strain) and disease activity (i.e., morbidity, prescription medication use, health appraisal) 20 years later, alongside the significant mediator of depression and anxiety (also measured at 10 years).

### Cultural Moderating Factors

The BBFM provides a structure for examining cultural differences in the effects of the BBFM pathways. For example, Priest and Woods (43) found that disease activity (i.e., morbidity and prescription medication use) of Latino Americans was predicted by a more NFEC, mediated by greater biobehavioral reactivity (i.e., anxiety and depression); the same pathways were supported for a more negative intimate partner emotional climate. Interestingly, the authors tested nativity status as a moderator of the BBFM's pathways, finding a significant direct pathway between family emotional climate and disease activity for U.S.-born Latinos which was non-significant for foreign-born participants. These results suggest that non-intimate family relationships may affect the physical health of U.S.-born Latino adults in ways not fully explained by the study's measure of biobehavioral reactivity. The study's findings may also imply that acculturation and health behavior play important mediating roles in the model, in keeping with other research regarding nativity-influenced health differences [e.g., research on the immigrant paradox; (44, 45)].

More recently, Priest et al. (36) tested the BBFM with a sample of African American adult participants in the Midlife Development in the United States Milwaukee project, incorporating considerations of discrimination to test its influence on family emotional climate, defined as family support vs. family strain. First, the authors found that greater discrimination was associated with worse family support and greater family strain; second, worse family support served as a significant mediator linking increased experiences of discrimination to worse biobehavioral reactivity (i.e., worse self-rated mental-emotional health). It is noteworthy that the authors also found that lack of family support (but *not* family strain, nor intimate partner support or strain) was associated with decreased biobehavioral reactivity (i.e., better self-rated mental-emotional health). It is possible that the BBFM could be expanded to incorporate considerations of social determinants of health—with discrimination as a powerful example—which impact health via the influence of these contextual stressors on family functioning, and stress.

## Implementation of Biopsychosocial Training and Patient Care in Medicine Facilitated by the Biobehavioral Family Model

One challenge to implementation of the biopsychosocial model in medical training is the lack evidenced-based models that not only emphasize the importance of the interconnection of biological, psychological, and social aspects of health and illness, but also specify pathways and mechanisms by which these factors influence one another. Such a model lends credibility to the biopsychosocial approach and a common language and guiding model for teaching and clinical assessment and intervention. We will present below two applications of the BBFM in residency and fellowship training (Authors, in Family Medicine Residency, and Authors, in Child and Adolescent Psychiatry Fellowship).

## Application of the BBFM in a Family Medicine Residency Program

Though primary care training stipulates a focus on biopsychosocial and behavioral health education, these curricula frequently fail to cover family systems in sufficient depth. Family medicine is an example of a discipline whose trainees report educational deficits in regard to family systems, and often lack exposure to couples and family therapy (46). This is important because, while it is well-recognized that patient illness impacts the family, it is also the case that family relationships impact the patient's illness (and response to illness). Primary care training that uses a systemic orientation to teach about families and health highlights this circular, mutual impact of patient disease on family relationships, and vice versa. Specifically, systems theory—the basis for family systems—is distinct from ecological or contextual theory: while each promote consideration of nesting, hierarchical levels of human environments, the latter may emphasize linear pathways of influence between any two adjacent systems, while systems theory postulates recursive, mutual impacts of the levels on one another via complex interactions. Thus, a family systems orientation is imperative in primary care in order to view all patients biopsychosocially, in the context of their families and communities, continually influencing *and* being influenced by the systems within which they are nested [and which are nested within them, i.e. physiological systems; (47)].

The BBFM model, being empirically tested, provides evidence for the impact of family relationships on individual health. Thus, it offers compelling justification for understanding a patient's disease in the context of family relations, which then informs their diagnosis and treatment. This is especially powerful in primary care: applying the constructs of the BBFM in primary care settings facilitates a clearer understanding of family-health connections for a wide range of patients and conditions. Specifically, the BBFM used in primary care, (1) systemically contextualizes patient illness, (2) delineates specific targets for assessment, (3) directs interventions toward areas that are maximally effective, and (4) supports primary care trainees in achieving competency in the prior three areas (i.e., systemic, family-oriented thinking, assessment, and intervention). The model provides the theoretical, evidence-based framework needed for the necessary paradigm shift in primary care, and resident training, toward family systems. Presented here is one example of how the model may be applied in family medicine residency, a setting in which training is embedded within clinical care.

### Training

In order to promote a family systems paradigm, and the ability of residents to think systemically, it is necessary for family systems training to be embedded within resident education broadly, rather than isolated as part of a single course or rotation (48). Educators teaching family-oriented care also require a “translation process,” and often must re-language complex relational and systemic concepts into a language familiar to, and easily understood by, physicians. The BBFM can help to achieve both aims: first, to guide the organization of resident psychosocial training, and to teach, demonstrate, and clarify why families are important for the work of primary care physicians. Second, the model serves as a clear, pragmatic map for educators engaged in such translation processes, as they interpret complex close relationships into case-specific content that is applicable to, and by, resident learners.

#### Foundation in BBFM Concepts

First, BBFM-guided training can be achieved via didactic exposure to research evidence for the influences of family and stress on illness. To serve as one example: primary care trainees are often taught a variety of aspects of *health behavior change*, including (a) the impact of specific health behaviors as well as (b) interventions to promote patient motivation and behavior change. To revise curricula specific to health behaviors, the BBFM concepts can be translated and applied to teaching the mechanisms whereby social networks influence health behavior, and thereby impact health outcomes. In other words, an educator can translate “*family emotional climate and the mediating link to health via biobehavioral reactivity”* as “*family support and family strain impacting patients' stress reactivity and mental health,”* as well as their health behavior. To demonstrate empirical support for this connection, research substantiating the BBFM's pathways can be described, explaining links between family relationships, depression/anxiety, and smoking, alcohol use, exercise, and binge eating [e.g., (33)]. Lastly, basic health behavior change interventions that are familiar to residents can be discussed, and then expanded upon to a family-oriented, systemic perspective. For example, educators can review the importance of assessing patients' readiness for change [e.g., from a motivational interviewing approach; (49)], including how patient “stress reactivity” can stifle confidence and behavior change enactment (50). Then, residents can be taught how to assess whether patients' family relations are possible barriers to change or are sources of support for disease management, thus highlighting the impact of family members' own health behaviors for promoting (or impeding) patient behavior change. Finally, lecture-based teaching can include using case-based examples or even role play [methods likely to resonate well with adult resident learners; (51)] modeling how to invite a patient's family into a behavior change intervention, emphasizing the importance of leveraging this critically important support network for improving patient adherence. This type of practice facilitates learning that can then be applied in the clinical space. Overall, family-oriented primary care first requires the recognition of the importance of families to health, and the impact of illness on family process. The BBFM can aid in encouraging a view of family members as important, worthy stakeholders, *and* contributors to the clinical process—that is, families as both resources and, at times, rate-limiting steps to change.

In addition to incorporating family emotional climate into teaching about specific clinical topics relevant to primary care, the successful translation of the BBFM framework for residents may advance their ability to think systemically. In other words, learning specific examples of recursive associations between family relationships and patient illness via a longitudinal curriculum approach (52) will likely generalize to residents being able to think biopsychosocially and in terms of hierarchy, responsivity, boundaries, and feedback loops (rather than merely a linear cause-and-effect relationship). Once the links between families and health are taught conceptually using examples, the content needs to be further mastered in the context of clinical application. The use of experiential training can ensure trainees recognize how families impact a patient's health, are able to assess relational process, and can intervene systemically.

Last, the application of the BBFM can be extended to residents' introspective training in order to increase their understanding of their own experiences of health and illness, and thereby increase their empathy for patients.

### Perception

The BBFM is useful in guiding residents' observation and perception of family relational process as it relates to a patient's disease or disorder, as well as guiding assessment of the psychosocial impact of illness on the patient and their family. One way the model aids resident perception is via use of a checklist with behavioral examples of the BBFM dimensions. This type of checklist can be used to facilitate residents' observations of family emotional climate in a patient's family. Such guides for observation should use terminology familiar to physicians in order to remove a mental leap doctors need to make to focus on family functioning. For example, if working with a patient and a family member, emotional climate becomes best conceptualized as *strain* (conflict, inconsistency, neglect) vs. *support* (openness, reliability, warmth, affection) in the dyad. Residents can also be guided to observe for power dynamics in the dyad, noting who is responsible for decision-making, or how the dyad negotiates shared responsibilities (e.g., parenting, housework, financial planning). Directing the resident to observe the qualities of this relationship ultimately increases the depth of their observation, and may facilitate residents moving to direct assessment.

### Assessment

Though the BBFM is not a therapeutic model, it is a well-defined theoretical foundation that can support therapeutic skill in primary care, beginning with assessment. For example, the BBFM-informed observational checklist described above can also be used as a map for screening and interviewing. First, beyond listening for cues regarding relationship quality, or observing family relationships in the exam room, physicians can be taught to ask basic questions to gather key information regarding support and strain among family members. As the BBFM conceptualizes both adaptive and maladaptive family functioning, it can guide trainees to assess both positive and NFEC, including praise vs. criticism, adaptability vs. passivity, flexibility vs. rigidity. Residents can also draw out patient-family member dyads and invite them to talk together about how they understand the doctor's recommendations, how they are working together to achieve treatment adherence, or how they would like to receive support from one another for lifestyle changes. To assess *responsivity*, physicians can verbally affirm observations of within-family empathy, ask about experiences of denial, secrecy, or isolation in the face of a new serious illness, or assess family-level emotion dysregulation due to worsening disease. The structural dimensions of family emotional climate can also be translated into patient assessment in primary care. *Proximity* (i.e., connectedness, caring, empathy, knowing what a family member is experiencing) can involve physicians asking patients about how they share their emotions with their family, or how they show one another affection. *Generational hierarchy* (i.e., power dynamics) can be evaluated during well child visits, demonstrated by parents' limit-setting or co-parenting. Maladaptive couple hierarchy is another possible area to assess, including power imbalances within a romantic relationship, which may increase stress and thus contribute to a patient's worsening disease activity.

As primary care physicians provide the bulk of mental health care in the U.S. (53), they are well-positioned, and often well-trained, to assess for stress, depression, and anxiety. However, an expansion of this training is needed to facilitate an understanding of stress reactivity as a mechanism by which the family emotional climate may be tied to disease; i.e., whether a patient's family emotional climate (i.e., stress or strain) is contributing to depression or anxiety in the patient. Having assessed family emotional climate variables, the family-stress-disease links should become more apparent than when physicians solely assess mood. The care of postpartum mothers and their infants (a form of family unit commonly encountered in primary care) provides an example of how this may be applied. Residents can be trained to assess for parents' relationship quality and spousal support, and how this may impact the mother's recovery after childbirth and ability to provide newborn care. Residents can also assess attachment security in the parents' relationship, and associations with maternal/paternal-infant attachment—another evidence-based dimension of the BBFM. The closeness and safety of these relationships may be tied to the newborn's physical development and demonstrating expected milestones during infant well child appointments. Conversely, a resident can assess whether an infant with a complicated neonatal course creates additional stress with which new parents may struggle to cope; this may be independent of the risk of postpartum depression, which is regularly screened for in the primary care setting. This example also highlights the recursive nature of associations between family and health; namely, that not only can family relationships impact health via stress, but also that illness, and related distress, impacts family. Because causal effects are presumed to be reciprocal in the BBFM, the model can be a useful map for trainees to observe the impact of illness on the patient's emotion regulation (i.e., biobehavioral reactivity) and the patient's family's relationships. Though some families may react to worsening disease with acceptance, open discussion, connection, or agency, others may buckle with isolation, renewed rifts, withdrawal, or resentment (54).

Although genograms are not a part of the BBFM, they importantly extend the utility of the BBFM as a guiding model. Genograms may be used to enhance residents' reflective ability and increase their awareness of family-health connections through examining systemic patterns in their own families-of-origin related to health and healthcare. Residents can extend the recognition of these patterns to discuss how patterns of family response to illness may be connected to their own development as a physician, as well as how they approach the patient-physician relationship. Though infrequently taught in family medicine residency programs (55), the genogram can powerfully support the development of empathy, especially if first applied to oneself. Lastly, the use of a genogram in direct patient care to assess family patterns can be taught in conjunction with the practice of gathering family health history, a critically important activity for documenting patients' disease risk (56).

The assessment of a patient's family emotional climate provides key insight into whether relationships may be contributing to the patient's disease or disorder, and if so, by what means: e.g., interfering with vs. supporting health-promoting behaviors, or contributing to disease activity through stress pathways or emotion dysregulation vs. soothing or modulating stress. Boosting physicians' abilities to observe relational processes first, and then linking them to health and illness via the pathways of the BBFM, increases the practice of thinking systemically as well as considering alternate aspects for intervention, aside from a limited focus on the “usual suspects” (e.g., an individual patient's medication adherence, diet, exercise).

### Intervention

Interventions used with patients and their families in primary care must first be organized around time considerations, with a priority on brevity. However, brief interventions need not sacrifice a family systems orientation. The BBFM also helps locate the most efficient route to effective brief intervention.

In training residents to intervene, it can be helpful to first focus on developing basic family interviewing techniques (57). The use of reflection statements can first serve to validate patient and family experiences, while the successful use of reframing statements can systemically shift a family's paradigm toward understanding their relational process and its influence on family members' well-being. As described above, the BBFM is designed to encompass aspects of relational functioning that promote resilience, as well as areas of vulnerability. It is thus helpful in identifying strengths, a core interviewing skill that can highlight what is working within the family, rather than exploring deficit alone. Example questions residents can ask to solicit the above include, “who else knows you are struggling?” and, “how do they support you taking care of yourself?”; these may help patients understand the power of their social network. This also leads the physician to explicitly assess who is supporting the patient, and who may be undermining them, which facilitates the active inclusion of supportive family members to strengthen those ties. In other words, this strategy identifies patients' social supports, but also whom, in the family, it would be most useful to engage in primary care visits. The first step in family-centered interventions is to determine who in the family would be most important to involve in care, either to mitigate negative/maladaptive relationship effects or to facilitate more supportive, adaptive ones.

Recruiting supportive family members to attend appointments, and meaningfully engaging them in the patient visit, adds a powerful, but brief, intervention to the physician's therapeutic toolkit. Patients may be more likely to understand treatment recommendations, and more apt to discuss difficult topics with their provider, when a support person joins their visits (58). Patients can also be encouraged to disclose worsening depression or anxiety to supportive family, and to open up to safe, empathic family members who can provide warmth and decrease isolation. We find that family members often intuitively sense the patient's distress or disease may be worsening but may be unsure how to offer their help. We therefore encourage physicians to directly reflect this possibility to the patient, and suggest a family pattern of dancing around issues of privacy and sensitive health issues, while simultaneously wanting more closeness. Family interviewing techniques applied during conjoint medical visits can include the resident assessing patient and family health beliefs (and whether they're aligned), cultural influences on health behaviors, family members' fears regarding the patient's disease, family members' perceptions of the patient's coping with a new diagnosis, or the impact of an illness on the family's functioning (59).

Family member attendance also necessitates a shift in point of care interventions to be intentionally more family-oriented. Brief family-oriented interventions in primary care can be easily adapted from existing interventions commonly used to promote patients' behavioral health. For example, motivational interviewing [frequently considered a powerful approach to shifting health behavior in primary care settings; (49)] can be adapted to be relational (60) via including the family in brainstorming ideas for change, assessing the family's support for behavior change, assessing family members as potential barriers to change, and scaling the family's buy-in and confidence in the intended change. Collaborative treatment planning with patients—discussing pros and cons of treatment options, developing next steps that reflect patients' values, etc.—can be easily shifted to engage family members in the process, thereby enhancing the likelihood of adherence and success (59). Finally, the BBFM, by promoting specificity and efficiency, may facilitate current movements toward single session mental health care in the context of primary care [e.g., (61)].

## Application of the BBFM in a Child and Adolescent Psychiatry Fellowship Program

In the United States, general psychiatry residents are typically introduced to a systemic biopsychosocial model of psychiatric disease, but are not allowed or encouraged to practice this way. However, child and adolescent psychiatry is much more cognizant of developmental, family, and socio-cultural contributions to child and adolescent psychiatric disorders, and thus there is a natural appreciation of the biopsychosocial model. However, there is a paucity of explanatory models that demonstrate how these complex processes interact to the benefit or detriment of the child's development and emotional functioning. Most urgently, child and adolescent psychiatry (CAP) fellows need a way to understand the child and adolescent in the developmental and relational context of their family, since the family is the most impactful, for better or worse, social context of the child. The BBFM is a model that can serve as a comprehensible map with which to navigate the complexity of family influence on the child and adolescent. We have developed a training module for our child and adolescent psychiatry fellows using the BBFM to provide basic conceptual tools for assessing and intervening with family relational process in order to support child and adolescent treatment goals.

The “Family Relational Assessment and Intervention” training protocol is devised of an annual 5-h didactic introduction to the basic principles of family systems theory and practice, followed by a biweekly 2-h clinical application seminar occurring over 6 months. There are two supervisors, a child, adolescent and family psychologist and a child and adolescent psychiatrist, working with 3 first year CAP fellows and 3 second year CAP fellows. In the didactic sessions, we contemplate the complex meanings and functions of “family.” We introduce the essential assumption of reciprocity of effect between individual and family levels of experience and behavior. We introduce child developmental staging via Eric Erikson's (62) Eight Stages of Man and Josephson's family psychodynamic developmental approach (63). We elucidate how these developmental stages shape family stages of development (64). Finally, we examine cultural aspects of family function, and incorporate the consideration of the vulnerability of families of minority and disadvantaged status. Broadly, we work to establish that a competent child and adolescent psychiatrist must be skilled in understanding and working with families. We help them understand that, more often than not, when a child or adolescent is struggling despite the best of treatment intentions and delivery, family challenges are often at the root, and must be addressed. And just as CAPs need to utilize their skills in individual psychotherapy modalities to help struggling patients get moving again, they can and must utilize family assessment, and intervention skills to help struggling families get unstuck to the benefit of their patients.

We introduce the BBFM as a map devised of specific dimensions with which to focus assessment of family function in relation to the child. We introduce the BBFM model as described above. However, we refer to it as the “Biobehavioral Family Model of Vital Signs” in order to emphasize that the dimensions described are essential to be observed and evaluated in any psychiatric evaluation of a child or adolescent. We renamed the technical term “proximity” to “care and connection” and “hierarchy” to “parental authority” because those are more familiar concepts but synonyms for the original terms. We translate our customary family systems terminology into terms which are more familiar and comfortable for medically trained residents. Overall, this helps CAP fellows to be able to understand not just that a family is dysfunctional, but how they are struggling and how one might intervene to help them. We do not use the term “systemic” but instead point out and help them observe the mutuality of causal effects of family member interactions, and the impact of sequential patterning of interactions. Our training program teaches and requires “biopsychosocial” psychiatric evaluations as a basic format, and we incorporate a BBFM evaluation to help fellows characterize their observations and construct an accurate “family relational” formulation which incorporates the aspects of the BBFM dimensions that are contributing to the child's difficulty.

The clinical application of the Family Relational Assessment and Intervention module occurs in the form of group supervision, live and video recorded. In order to assist the fellows in learning to observe and perceive patterns of family interactions so as to characterize the BBFM aspects of family function, we use a standardized “Family Process Assessment Protocol” (65). The Family Process Assessment Protocol was originally developed for Wood's family and child asthma laboratory-based research described above. The results of these studies demonstrate the validity and utility of this assessment device in characterizing dimensions of the BBFM (66). The first year CAP fellows conduct these assessments and write BBFM informed assessment evaluation. The second year CAP fellows observe these assessments and contribute to the family relational formulation and treatment planning. On alternate meetings the second year CAP fellows invite families in treatment for live group supervision and discussion using a one-way mirror. In addition, if schedules require, the supervisor and fellow meet at an alternate time and record assessments and interventions in order to bring them to the bi-weekly meetings to review and discuss.

### The Family Process Assessment Protocol

Child and adolescent psychiatry fellows identify families of children whom they think would benefit from the family clinical assessment. They recommend to the families that they come to the clinic to “participate in a series of discussion tasks in order for us to learn more about how your family works and plays together.” The fellow explains that “this will help us to better understand you and your child, and therefore learn how best to help your child.” They further explain that “we work in consultation teams, so we will be observing your discussions from behind a one-way mirror.” The supervisor and non-interviewing fellows observe from behind the mirror as the supervisor points out patterning of interactions and engages the fellows in describing what patterns they are observing. While there is no intentional intervention provided during these assessments, we focus on the “family vital signs” as a framework for what specific dynamics to observe and to help fellows consider possible future interventions. We also help fellows appreciate, through observing the FPAP, that any guided, supportive family discussion can be, in and of itself, a therapeutic family intervention.

The first-year fellow treating the child conducts the protocol, presents the instructions for each task, and returns to behind the mirror to observe the family's discussions. The discussion tasks each last 5–10 min and are designed to elicit a range of emotionally tinged interactions. The fellow presents the discussion task to the family explaining that he or she leaves “in order not to distract the family from their own discussions but will be observing.” Tasks: (1) invite the family to build a house of cards; (2) have the child present a difficulty he or she is having to the family for their help; (3) have the child tell the family the story of something that currently, or in the past, made them sad; (4) request that the parents and child discuss and resolve a previously identified disagreement; (5) have the parents (if there are two) discuss and resolve a previously defined disagreement; (6) ask family members to go around, one at a time, and say what they like best about each family member, and about the family as a whole.

At the end of the family discussions, the supervisor and fellow construct brief feedback regarding strengths observed and one or two ways of relating that need to change in order to support the child's recovery. The fellow then provides this feedback to the family. For example, based on the BBFM dimensions, the fellow might note, “your family is clearly warm and supportive of one another (family emotional climate), and clearly your child (the patient) looks to you parents for reassurance and guidance” (secure attachment). “But mom and dad unintentionally undermine one another's authority because they have very different parenting styles” (weak parental alliance). “This allows your child to ignore your instructions and be defiant (for a child with behavior problems).” Often, if invited to share their own observations, family members will themselves identify these relational challenges even before the fellow provides feedback. The fellow invites further questions from the family and explains that he/she will assist the family in working on these changes and address other relationship needs in subsequent intervention sessions. The supervisor provides ongoing supervision of the future family relational interventions. The patterns observed during the family protocol help the fellow to construct the BBFM evaluation, using the BBFM model and definitions (above) to develop his or her formulation of the family's contribution to the child's strengths and difficulties, and to devise recommendations for intervention. The supervisor provides instructive comments, edits and suggestions for the BBFM evaluation.

Once the first-year fellows become proficient in using the BBFM as a map guiding the observation of “family vital signs,” they naturally begin to observe other family relational patterns that are relevant to the child's difficulty, or that can be tapped to support the child's improvement. The first-year fellow generally continues intervention with the family that s/he brought into the family assessment protocol, while the family supervisor continues supervision on the case. In their second year, fellows identify at least one additional case in which they seek live supervision from one of the supervisors. These cases are live supervised or recorded and are brought to the group clinical supervision to provide clinical material for discussion of strategies for intervention. Basic intervention strategies are taught targeting dimensions of the BBFM. For example, (1) facilitating positive *family emotional climate* by redirecting negative interactions; (2) interrupting interactions reflecting poor parent-*child proximity or connection*, as when parents are not listening to the child and the child is escalating, by asking that the parent slow down and listen to the child and then reflect back to the child what the parent is hearing the child convey; (3) reframing highly reactive and hostile behavior as defensive and reflecting sadness and feelings of dismissal or rejection (signs of *insecure attachment)*; (4) redirecting parents if they show *poor parental alliance* by conveying opposite messages to the child by asking them to talk with one another to sort out the different ways in which they are responding to the child; (5) interrupting, and pointing out interactions in which a parent is undermining the other parent's instructions to the child, i.e., *weak parental hierarchy*. These examples reflect family systemic interventions, but are not exclusive. In general, these interventions interrupt and redirect maladaptive (based on BBFM concepts) patterns of interaction to those that are consistent with the positive aspects of the BBFM model. There are many other models of family systemic intervention that are consistent with the BBFM (67).

There are many challenges to the success of this approach to training in family assessment and intervention. It is often difficult to schedule everyone in the household to come in at the same time (we typically start with both parents or caregivers, if they are living in the house, and children over 5 years of age living in the house). If the group supervision time slot cannot work, we offer another appointment and the supervisor provides direct supervision of the assessment and/or ongoing therapy. These sessions are then recorded and brought to group supervision.

Another challenge is in engaging fellows in an area in which they are quite unfamiliar and uncertain. It is helpful to avoid language that is unfamiliar and to translate concepts into terminology that they find comfortable and consistent with their training. The most important inducement is for the fellows to be able to better understand the “how” and “why” of complex cases, and feel increased self-efficacy in being able to help untangle some of the challenging psychosocial contributors to a child's dysfunction. To support fellows in learning these complex skills, the supervisor must be readily available to provide direct support to the trainee throughout the learning process.

The clinical context of the training can be a challenge as well. Training often occurs in clinics or service centers where services are provided by a variety of professions, including social workers, psychologists, counselors, and nurse-clinicians. In these settings, child and adolescent psychiatry fellows are in high demand to provide psychiatric evaluations and medication recommendations and follow up. Thus, time must be protected so that they can carry psychotherapy cases that would appropriately require family assessment and intervention. In our training site, our CAP fellows frequently conduct Family Process Assessment Protocols for the patients of other clinicians who have requested a medication assessment from them. They explain that it is important to evaluate the patient in the context of his or her family, and they propose a family assessment. However, if the clinician explains that they have assessed the family and are currently engaging the family in the child's therapy, the fellow will conduct the psychiatric evaluation and treatment, relying on the clinician's report of family functioning. Alternatively, if the clinician agrees and the fellow conducts a family assessment, the clinicians are provided with a copy of the BBFM evaluation along with the usual psychiatric assessment and treatment recommendations. Often we will invite the clinician to observe the Family Process Assessment Protocol. This serves to build relationships of trust among the fellows and the clinicians, and it provide clinicians with opportunities to expand their perspective on the role family relations play in child emotional, psychological, and behavioral disorders. We also are developing a family consultation service for our clinicians, where we will conduct a Family Process Assessment Protocol for them, so that they can have that information to inform their work. When we have done this in the past, collaborations often develop, with the clinician focusing on individual intervention and the fellow focusing on family relational work.

This year the coronavirus pandemic precluded in-person family assessments and interventions. We adapted to this challenge by employing telemedicine technology. In order not to overwhelm the families and the fellows with the intensity of group online observation of telemedicine interviews, we limited the assessments and interventions to one fellow and the supervisor. We recorded the session, with family permission, and used the recordings in the group supervision seminars to observe patterns of interaction as they related to the child's problems, discussed the interventions made and the effects they had, and planned for additional interventions for future sessions. There are certain advantages to this method, some of which may be adapted to telemedicine-based child and adolescent behavioral health: (1) it is easier to schedule the families in their homes; (2) it is very graceful to make on the spot supervisory suggestions via the private chat option in telemedicine technology; (3) we can review the recorded session in group supervision, allowing the flexibility to pause a session in order to observe, reflect, and understand the therapy in the moment, which is an efficient teaching strategy. In addition there is the opportunity to ask the fellow (or supervisor) about his or her purpose in given interventions. There are also significant disadvantages to the telemedicine approach. Most prominent are challenges in technology in the home which can cause degraded audio or visual quality; keeping the family within view; the personal awkwardness of seeing oneself on the screen; by the relatively impersonal feel of the method compared to in-person therapy; and by the limits it imposes on the clinician to be able to control escalating sequences of family conflict, which are more easily interrupted and diverted in person (the technology prioritizes the current speaker and it can be difficult for the clinician to interrupt).

The take-home message is that challenges to teaching family assessment and intervention in the context of child and adolescent psychiatry training can be overcome with strong supervisory commitment and close connection to each fellow, and flexibility and variability in formatting the experience. Although working within health care systems that appear to primarily value the child and adolescent psychiatrist as “prescribers,” the children, adolescents, and families we are asked to help are often the most impaired and in need of comprehensive assessment and treatment. At his or her core, the child and adolescent psychiatrist needs to be able to formulate a deeper understanding of what is happening in a child's life that results in impaired functioning. In this context, training and embracing the BBFM as a framework to assess and intervene with struggling families provides CAPs both with a way to help these most impaired of children and families, as well as a way to reclaim a broader role and skillset.

## The Leading Edge: Future Directions for the BBFM

The BBFM's specified pathways provide multiple avenues for future research, training, and clinical directions. For example, including temporal dynamics and developmental trajectories in the model would enrich and extend its scope and predictive power. This would be useful from a life course perspective, as well as for modeling disease trajectories over a period of aging. The BBFM may also be of heuristic value in extending testable theory of family and individual resilience, which would support key prevention strategies. The inherent culturally flexible constructs of the BBFM makes it useful for examining causes of health disparities, and potentially to discover family patterns which may improve contributing factors including patient disease self-management. Additional research is needed to identify how societal contextual stressors contribute to the psychobiological mechanisms linking family emotional climate and disease activity identified by the model. Ultimately, research and successful applications of the BBFM could inform policy to improve the lives of families.

Though Engel's BPS model presented a critical advance for medicine, its application has been limited by its lack of specificity, constraining the model's ability to guide research and clinical practice. Further, the BPS model does not provide a well-defined language that functions across disciplines, which is necessary to facilitate integrated care. The BBFM, however, provides a specific biopsychosocial conceptualization for the study and treatment of illness in families, while acknowledging both the protective and negative impacts of family process for health. The empirical evidence supporting the model's theoretical underpinnings ultimately lends support for Engel's BPS approach. The identification and operationalization of specific, testable constructs, and mechanisms of effect provides a guide for family-based research, training, and clinical care. The continued application and modification of the BBFM will further serve to enhance the implementation of BPS theory in medicine. Our hope is that in presenting this model, others will find it similarly useful in developing innovative research, training opportunities, and practice approaches. In addition, we recommend that the BBFM serve as a prototype for other multi-level, systemic, biopsychosocial modeling.

## Author Contributions

BW and SW contributed to the conception and outline of this review, and wrote the initial drafts of the manuscript. SS and TN contributed to the full draft and wrote sections of the manuscript specific to the training programs. All authors contributed to manuscript revision, and approved the final submitted version.

## Funding

This work was supported by the National Heart, Lung, and Blood Institute, funding awarded to BW (R01HL123609).

## Conflict of Interest

The authors declare that the research was conducted in the absence of any commercial or financial relationships that could be construed as a potential conflict of interest.

## Publisher's Note

All claims expressed in this article are solely those of the authors and do not necessarily represent those of their affiliated organizations, or those of the publisher, the editors and the reviewers. Any product that may be evaluated in this article, or claim that may be made by its manufacturer, is not guaranteed or endorsed by the publisher.
